# Changes in Membrane Plasmalogens of *Clostridium pasteurianum* during Butanol Fermentation as Determined by Lipidomic Analysis

**DOI:** 10.1371/journal.pone.0122058

**Published:** 2015-03-25

**Authors:** Jan Kolek, Petra Patáková, Karel Melzoch, Karel Sigler, Tomáš Řezanka

**Affiliations:** 1 Department of Biotechnology, University of Chemistry and Technology Prague, Prague, Czech Republic; 2 Institute of Microbiology, Academy of Sciences of the Czech Republic, Prague, Czech Republic; National Taiwan University, TAIWAN

## Abstract

Changes in membrane lipid composition of *Clostridium pasteurianum* NRRL B-598 were studied during butanol fermentation by lipidomic analysis, performed by high resolution electrospray ionization tandem mass spectrometry. The highest content of plasmalogen phospholipids correlated with the highest butanol productivity, which indicated a probable role of these compounds in the complex responses of cells toward butanol stress. A difference in the ratio of saturated to unsaturated fatty acids was found between the effect of butanol produced by the cells and butanol added to the medium. A decrease in the proportion of saturated fatty acids during conventional butanol production was observed while a rise in the content of these acids appeared when butanol was added to the culture. The largest change in total plasmalogen content was observed one hour after butanol addition i.e. at the 7th hour of cultivation. When butanol is produced by bacterial cells, then the cells are not subjected to severe stress and responded to it by relatively slowly changing the content of fatty acids and plasmalogens, while after a pulse addition of external butanol (to a final non-lethal concentration of 0.5 % v/v) the cells reacted relatively quickly (within a time span of tens of minutes) by increasing the total plasmalogen content.

## Introduction

Renewable butanol, which currently attracts the attention of many researchers, might become a prized fuel extender or a valued chemical substance if obstacles to its fermentative production, namely low final concentration, yield and process productivity, could be overcome. The most typical solventogenic species, *Clostridium acetobutylicum*, forms butanol by acetone-butanol-ethanol (ABE) fermentation of sugars. In batch culture, exponentially growing cells produce organic acids, which lower the pH of the medium. As the culture enters stationary phase, metabolism of the organism changes, carbohydrates and a proportion of preformed organic acids are converted into organic solvents, mainly butanol and acetone, and the cells sporulate [1]. However, some solventogenic clostridia, e.g. *Clostridium beijerinckii*, *Clostridium pasteurianum*, and others, may convert substrates into butanol by an ABE process that differs from the classical pattern and in which acidogenic and solventogenic phases overlap; butanol production starts during exponential growth, produced acids need not be completely transformed into solvents and the ratio between produced solvents (butanol:acetone:ethanol) differs from the typical one (6:3:1) [2,3]. The strain used for this study, *Clostridium pasteurianum* NRRL B-598, excels in oxygen tolerance, is not prone to degeneration (loss of solvent production), its physiology has been described [4–7] and its genome has been published recently [8].

The mechanism of butanol toxicity is related to the hydrophobic-hydrophilic nature of this compound. The primary effects of this molecule, studied in *C*. *acetobutylicum*, appears to be disruption of the phospholipid component of the cell membrane [9], variations in membrane composition and fluidity [10] and an increase in the proportion of saturated fatty acids and the mean acyl chain length of fatty acids in the cell membrane. Changes in membrane fluidity in the presence of butanol results in destabilization of the membrane and disruption of membrane-linked functions.

Several mechanisms of membrane adaptation to a high concentration of butanol are possible—a change in the degree of saturation of fatty acids, cis/trans isomerization of unsaturated fatty acids, including changes of a double bond to a cyclopropane ring, and changes in the composition and dynamics of phospholipids [10,11,12]. The most important of these mechanisms is the composition and turnover of phospholipids, which have only been studied in detail in aerobic bacteria [12,13].

Fatty acids in complex lipids of the obligate anaerobe genus *Clostridium* have been thoroughly described [14]. The species *C*. *pasteurianum* has been studied much less than the important butanol producer *C*. *acetobutylicum* or the pathogenic *C*. *tetani*. The major fatty acid found in *C*. *pasteurianum* was palmitic acid. Also, changes in membrane fatty acid content during ABE fermentation and butanol-chalenge cultivation were measured previously in type strain *C*. *pasteurianum* ATCC 6013 [15]. The most abundant alk-1-enyl chain commonly found in position *sn*-1 of plasmalogens was c-17:0 (*cis*-9,10-methylene-hexadecanoic acid) [16]. However, it should be noted that out of the many studies on lipids found in this genus, only Johnston and Goldfine [16] have identified alkenyl chains in *C*. *pasteurianum*.

A similar situation occurs with complex lipids. The most thoroughly studied strains are pathogens such as *C*. *tetani* [17], *C*. *botulinum* [18], some other strains such as *C*. *novyi* [19] and *C*. *psychrophilum* [20], and the butanol producer *C*. *acetobutylicum* [21]. The content of polar lipids in *C*. *pasteurianum* was studied several decades ago [16] and thus the data cannot be easily compared with results obtained by modern methods, especially by soft ionization mass spectrometry (see the above references). Based on published data [1], we can assume that the major polar lipids in *C*. *pasteurianum* are likely to include phosphatidyl ethanolamine (PE), phosphatidyl glycerol (PG), phosphatidyl serine (PS), and cardiolipin (CL, i.e. *bis*-phosphatidyl glycerol) in both diacyl and plasmalogen forms, since the species belongs to “related organisms in cluster 1, and many of these organisms are capable of producing alcohols and solvents”[14]. The main goal of this study is to describe changes in membrane phospholipid (plasmalogen) composition and concentrations in *C*. *pasteurianum* NRRL B-598, elicited by both solvent formation and sporulation (cell cycle), using lipidomic analysis by high resolution electrospray mass spectrometry (ESI-MS) on an Orbitrap mass spectrometer. In addition, changes in cell fatty acids and plasmalogen contents after butanol addition to solvent non-producing cells of this strain are described for the first time.

## Material and Methods

### Microorganism and culture conditions

The solvent-producing strain *C*. *pasteurianum* NRRL B-598 (for its draft DNA sequence see http://www.ncbi.nlm.nih.gov/bioproject/231609), maintained as a spore suspension, was used throughout this study. The strain was grown at 37°C in a 7 l laboratory BIOSTAT B bioreactor (B. Braun Biotech Int., Germany) with 4 l modified TYA medium [22] (containing g/l: glucose 20; yeast extract 2; tryptone 6; KH_2_PO_4_ 0.5; ammonium acetate 3; MgSO_4_.7H_2_O 0.3; FeSO_4_.7H_2_O 0.01), at 200 rpm agitation and without pH control. The bioreactor was inoculated with 10% v/v of a 18-h culture grown from spores. Two liquid samples were taken during fermentation for substrate, biomass and product analyses, and for lipid extraction and isolation. For extraction of lipids, biomass samples from cultivation broth (500 ml) were harvested by centrifugation (8.000 x g for 10 min. at 4°C). After washing with deionized water, biomass was resuspended in 20 ml of deionized water and lyophilized using a Modulyo freeze dryer (Thermo Electron Corporation, USA).

### Cultivation with butanol challenge

Cultivation with butanol addition was carried out under the same conditions as described above. Six hours after the beginning of culture, pure 1-butanol (Sigma-Aldrich, Prague, Czech Republic) was added to the bioreactor, to a final concentration of 0.5% (v/v).

### Growth measurement

Cell concentration was gravimetrically determined as biomass dry weight. The dry weight of biomass was measured from 2ml samples taken during fermentation, centrifuged (10.000 x g for 5 min.), washed with deionized water, and dried at 105°C for two hours. Dry cell biomass was weighed after cooling to room temperature in a desiccator and the remaining supernatant was used for substrate and product analyses. Results represent average values of three measurements.

### Substrate and product analyses

The concentrations of glucose, butyric acid, acetic acid, acetoin, butanol, acetone and ethanol were measured in the microfiltered (0.2 μm nitrocellulose membrane) supernatant of culture broth by HPLC (Agilent Series 1200 HPLC; Agilent, Spain) using a Polymer IEX H^+^ column (Watrex, Czech Republic) and refractive index (RID) detection (Agilent Series 1200 Refractive Index Detector; Agilent, Spain). Isocratic elution with a mobile phase of 5mM H_2_SO_4_ used the following parameters: stable column temperature 60°C; mobile phase flow rate 0.5 ml/min; injection sample volume 20 μl.

### Measurement of fermentation gases

During fermentation, gases from the headspace of the bioreactor were collected in 80 l gas-tight Tedlar^®^ bags with a valve, and the total gas volume was measured at the end of fermentation using a gas pump. Gas composition was determined by the gas chromatograph (HP 6890 Series GC System) equipped with an Agilent CHROMPACK CP capillary column (column parameters: 50m×0.53mm; film thickness 15μm) and a thermal conductivity detector. GC parameters were the following: amount of sample, 500 μl; inlet temperature, 150°C; pressure, 60 kPa; flow in the column, 7.8 ml/min; carrier gas, nitrogen.

### Lipid extraction and isolation

All chemicals were purchased from Sigma-Aldrich (Prague, Czech Republic). The extraction procedure was based on the method of Bligh and Dyer [23], except that 2-propanol was substituted for methanol, since isopropanol does not serve as a substrate for phospholipases [24]. The alcohol-water mixture of the frozen cells was cooled, one part of chloroform was added and the lipids were extracted for 30 min. Insoluble material was sedimented by centrifugation and the supernatant was separated into two phases. The aqueous phase was aspirated and the chloroform phase was washed three times with two parts 1 M KCl each. The resulting chloroform phase was evaporated to dryness under reduced pressure.

### LiAlH_4_ reduction and separation of 1-alkenyl-sn-glycerols by TLC

Total lipids were reduced with lithium aluminium hydride (LiAIH_4_) by a modified procedure described by Wood and Snyder [25]. Briefly, LiAlH_4_ (15 mg in 3 ml of diethyl ether) was added to 3 mg of total lipids in 1 ml of ether. The reaction mixture was refluxed for 1 hour and 3 ml of distilled water, 3 ml of 4% acetic acid, and 3 ml of diethyl ether were added. The diethyl ether phase was then removed, evaporated to dryness and separated by TLC (PLC Silica gel 60, glass plates 20 x 20 cm x 2 mm, Merck, Prague, Czech Republic) using a mixture of diethyl ether-30% aqueous ammonium hydroxide (99.75:0.25 v/v). Band visualization was carried out with 0.2% 2',7'-dichlorofluorescein in ethanol and the appropriate band (R_f_ 0.5, i.e. 1-alkenyl-*sn*-glycerol) was scraped off from the preparative plates, eluted by a diethyl ether-water (99.5:0.5 v/v) mixture, evaporated and analyzed by MS.

### Lipidomic analysis by high resolution ESI

A high resolution hybrid mass spectrometer LTQ Orbitrap Velos (Thermo Fisher Scientific, Prague, Czech Republic) was used. ESI–MS analysis was performed in the positive ion mode. MS spectra were obtained by the FT mode and were acquired with target mass resolution of R = 30.000 at *m/z* 400 (lock mass 413.6662 Da). The ion spray voltage was set at -2500V and the scan range of the instrument was *m/z* 200–1500. Nitrogen was used as a nebulizer gas set at 18 arbitrary units (sheath gas) and 7 arbitrary units (aux gas). Helium was used as a collision gas for collision-induced dissociation (CID) experiments. A CID normalization energy of 35% was used for the fragmentation of parent ions. The MS/MS product ions were detected in the low resolution FT mode. Flow Injection Analysis (FIA) was used for sample introduction into the heated ESI (H-ESI) ion source. Pure acetonitrile was used at a flow rate of 150 μl/min. The H-ESI temperature was set to 250°C.

## Results

### Standard Batch cultivation

An initial glucose concentration of 20 g/l was chosen so as to be able to follow the changes in membrane plasmalogens of *C*. *pasteurianum*, during both acid and solvent production, spanning one complete sporulation cycle. The bioreactor was inoculated with an 18-h culture containing only vegetative cells. At the end of fermentation, numerous spores were released from mother cells. If the glucose concentration was higher, the spores would germinate and a second incomplete sporulation cycle would be started, as observed during previous experiments (data not shown).

**Fig 1:**
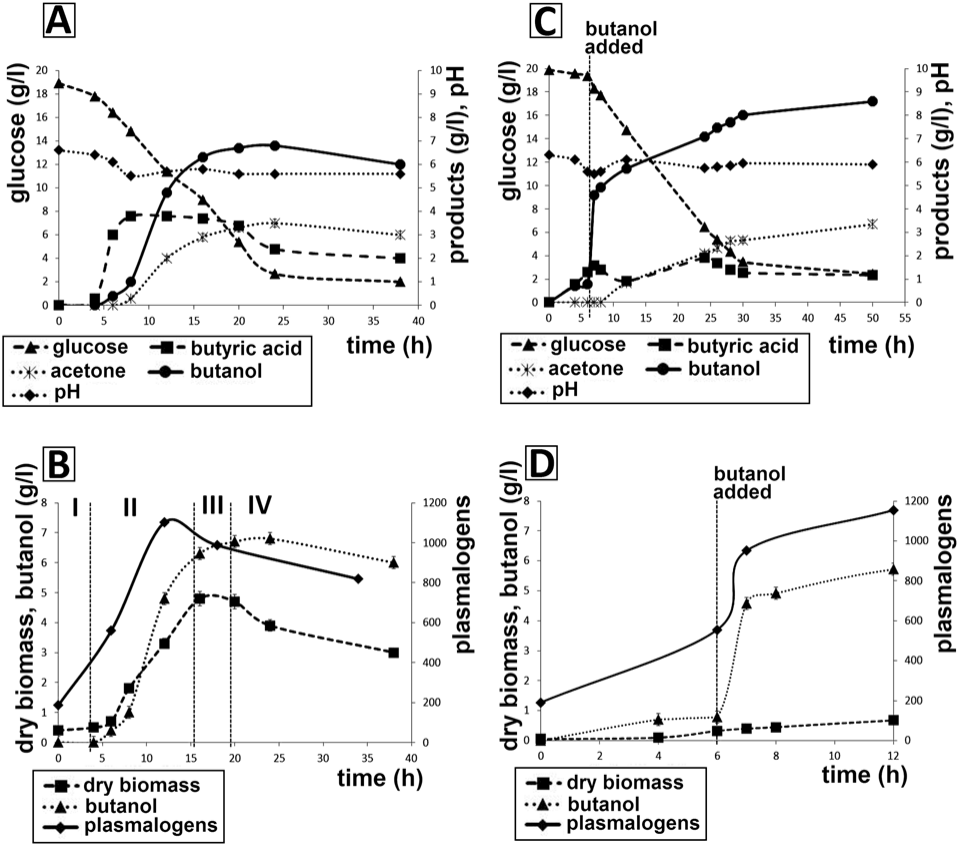
Main variables characterizing batch fermentation and butanol challenge cultivation of *C*. *pasteurianum* together with plasmalogen content. A,B—fermentation without added butanol, C,D—fermentation with butanol pulse addition (to a final concentration of 0.5% (v/v)), 6 h from the start of fermentation. Numbers I, II, III, IV correspond to growth phases. The abundance of total plasmalogens is given in the intensity of reconstructed ion currents (dimensionless value). The presented data are average from three replicates; standard deviations of substrate and products HPLC analyses did not exceed 5%.

The main indicators of batch fermentation, i.e. concentration of products and substrate, growth curve and pH, are shown in Fig. 1A and B. In addition to values presented in Fig. 1, acetic acid, acetoin and ethanol concentrations were also determined but they are not shown because they were formed in negligible amounts (ethanol, acetoin) or their concentrations did not change significantly (acetic acid). Both Fig. 1A and 1B unambiguously show that the production of butanol by this strain starts during the early exponential growth phase and follows that of butyric acid with only a small delay. Calculated fermentation parameters during individual growth phases, marked by Roman numerals (Fig. 1B), are shown in Table 1; the highest values for both butyric acid and butanol productivities were reached during the exponential growth phase, whereas glucose consumption rate was the highest during the stationary phase. This might be ascribed to sporulation, which is highly energy-consuming and culminated in this period. A decrease in butyric acid productivity during stationary phase can be explained by its moderate consumption, together with glucose; a decrease in butanol concentration during late stationary phase was caused by its evaporation and stripping by fermentation gas leaving the bioreactor. In total, 29 l fermentation gas were formed, comprising 37% v/v hydrogen and 63% v/v carbon dioxide.

**Table 1:** Fermentation parameters.

Growth phase (time interval in hours)	R_X_ *—growth rate (g/l/h)	P_B_ *—butanol productivity (g/l/h)	P_BA_ *- butyric acid productivity (g/l/h)	R_G_ *- glucose consumption rate (g/l/h)
I-lag (0–4)	0.03	0.0	0.08	0.28
II-exponential (4–16)	0.38	0.5	0.28	0.73
III-stationary (16–20)	-0.03	0.1	-0.08	0.90
IV-death (20–38)	-0.09	-0.04	-0.08	0.08

Average values are given for the individual periods. Growth phases are shown in Fig. 1B.

* The following calculations were used: R_x_ = ΔX/(V×Δt); P_B_ = ΔB/(V×Δt); P_BA_ = ΔBA/(V×Δt); R_G_ = ΔG/(V×Δt). V = working volume of bioreactor [l]; *Δt* = defined time period [h]; *ΔX*, *ΔB*, *ΔBA*, *ΔG* = change of the amount of biomass, butanol, butyric acid or glucose in the bioreactor during the defined time period, respectively [g].

### Butanol challenge cultivation

A final concentration of 0.5% (v/v) added 1-butanol in the butanol challenge experiment led to a sudden slowing of growth rate, which was followed by another increase in biomass concentration and butanol production. The butanol concentration increased considerably after butanol addition and its final concentration in the medium was higher than during standard batch cultivation. Addition of butanol led to a decrease in butyric acid production (Fig. 1C and D); pH and spore formation values were not significantly affected. Fermentation gasses (total volume 27.5 l) were of the same composition as during batch cultivation.

### Lipidomic analysis

Lipids were obtained from the lyophilized *C*. *pasteurianum* cells by extraction, according to Bligh and Dyer [23]. The major fatty acids were found to be palmitic and oleic acids, and the major plasmenyl chain was methyleneoctadecanal (c-19:0). Table 2 shows the clear dependence of fatty acid and plasmalogen content in total lipids on culture age, and on butanol addition in butanol challenge cultivations. The highest level of dimethylacetals, and therefore plasmalogen phospholipids, occurred in batch cultivation after 12 h, i.e. at the time when production of butyric acid was already constant (Fig. 1) and butanol productivity (0.5 g/l/h) was maximal (Table 1). During the butanol challenge cultivation we found a significant increase in plasmalogen phospholipid content immediately after butanol addition (Fig. 1). To determine how the FAs or plasmalogens are bound in polar lipids, we carried out a complex analysis of polar lipids. The most abundant molecular species in all 4 major phospholipids (PE, PG, PS and CL) was the combination of palmitic acid and a c-19:0 plasmenyl chain (see mass spectra in S1–S5 Figs). Detailed analyses revealed other polar lipids, especially glycolipids, in amounts lower than those of the above 4 phospholipids. As a part of further analyses, we therefore simplified the determination of total plasmalogen forms of polar lipids.

**Table 2:** Fatty acid and dimethylacetal composition in *C*. *pasteurianum*.

	Time (h)								
FAME+DMA^a^	0h	0h^b^	6h	6h^a^	7h^b^	12h	12h^b^	18h	34h
12:0	0.1^c^	0.1	0.1	0.1	0.1	0.1	0.0	0.1	0.1
13:0	0.8	0.8	0.7	0.8	0.8	0.6	0.6	0.7	0.8
14:0	5.0	5.2	4.7	4.9	5.9	4.8	4.8	4.9	4.8
c-15:0	3.3	3.4	3.1	3.3	2.7	3.2	3.2	3.2	3.4
i-15:0	0.2	0.2	0.1	0.1	0.0	0.1	0.0	0.3	0.4
15:0	1.7	1.8	1.6	1.7	1.8	1.7	1.7	1.6	1.7
16:1	8.9	9.1	8.4	8.5	4.5	8.5	8.3	8.4	8.6
16:0	55.3	54.1	51.5	51.2	55.8	39.7	47.6	45.7	49.8
c-17:0	2.4	2.5	2.1	2.2	1.2	1.9	1.9	2.3	2.6
17:0	0.7	0.7	0.5	0.6	0.8	0.4	0.4	0.6	0.5
18:1	4.8	5.0	7.1	6.8	3.4	8.7	8.1	7.6	6.9
18:0	2.4	2.4	2.5	2.3	4.2	2.9	2.9	2.4	1.8
c-19:0	0.6	0.6	0.5	0.6	0.2	0.6	0.6	0.5	0.6
16:0 DMA	1.5	1.5	1.8	1.7	2.7	2.0	1.9	1.5	1.1
16:1 DMA	0.8	0.8	0.8	0.7	1.1	0.8	0.8	0.8	0.8
c-17:0 DMA	2.3	2.3	2.6	2.5	3.8	3.0	3.0	1.9	1.6
c-19:0 DMA	9.2	9.5	11.9	12.0	17.1	21.0	14.2	17.5	14.5
**saturated (FAME)**	**66.0**	**65.1**	**61.6**	**61.6**	**69.4**	**50.2**	**58.0**	**56.0**	**59.5**
**unsaturated (FAME)**	**13.7**	**14.1**	**15.5**	**15.3**	**7.9**	**17.2**	**16.4**	**16.0**	**15.5**
**cyclo (FAME)**	**6.3**	**6.5**	**5.7**	**6.1**	**4.1**	**5.7**	**5.7**	**6.0**	**6.6**
**total DMA**	**13.8**	**14.1**	**17.1**	**16.9**	**24.7**	**26.8**	**19.9**	**21.7**	**18.0**

^a^ FAME—fatty acid methyl esters; DMA—dimethylacetals

^b^ Cultivation with the addition of butanol at 6th hour.

^c^ Standard deviations of all relative percent concentrations in this table are <0.13 as calculated from three replicates.

**Fig 2:**
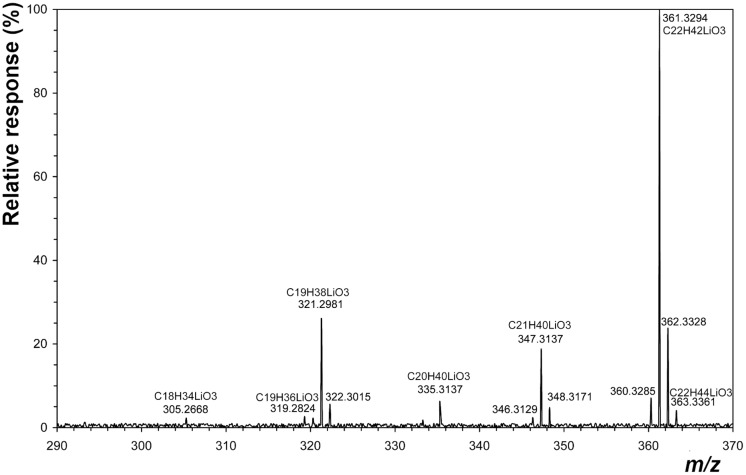
High resolution electrospray mass spectrum (ESI–MS) of plasmalogen alcohols (1-alkenyl-*sn*-glycerols) from *C*. *pasteurianum*. For experimental conditions, see ‘‘Material and Methods“. Numbers correspond to accurate mass values.

Our method is based on TLC separation and mass spectrometric identification of the 1-alk-1´-enylglyceryl ethers that are formed by LiAlH_4_ hydrogenolysis of phosphate and carboxylate esters of phospholipids [25]. TLC separation of the reaction mixture yielded a corresponding band of alkenyl glyceryl ethers, which was used to identify appropriate homologs by ESI MS (see Materials and Methods). Fig. 2 shows the mass spectrometry profile, which indicates that the major compound was c-19:0 glyceryl ether. These steps, i.e. reduction, separation and determination by ESI MS were performed in order to simplify the identification of plasmalogens. Of note is that one of the most abundant molecular species, c-p-19:0–16:0/16:0-c-p-19:0, involves 13 pseudomolecular ions. Based on this finding we therefore carried out a determination of all plasmalogens in the form of alkenyl glyceryl ethers.

## Discussion

Strain *C*. *pasteurianum* NRRL B-598, does not follow the typical ABE fermentation pattern, which was established for the most frequently studied strain, *C*. *acetobutylicum* ATCC 824. It differs from this strain especially in the early commencement of butanol production and a moderate re-consumption of previously formed acids. Its physiology, described in detail elsewhere [4–6], seems to be quite close to *C*. *beijerinckii* NCIMB 8052 [3]. In addition, the strain also differs from the type strain *C*. *pasteurianum* DSM 525 in its inability to consume glycerol, but resembles the type strain in the massive production of hydrogen.

Butyric acid and butanol cause stresses which mutually overlap, and elicit a complex response in the cell culture. For *C*. *acetobutylicum*, it was postulated that this mechanism includes regulation of cell membrane fluidity. Usually, changes in membrane fluidity are described as being based on an increase in the ratio of saturated to unsaturated fatty acids and a lengthening of fatty acid chains under stress conditions [9,26,27]. At variance with previous studies [9,10] we observed a decrease in the proportion of saturated fatty acids during conventional butanol production while a rise in the content of these acids appeared when butanol was added to the culture.

However, discrepancies regarding fatty acid formation under butanol stress during ABE fermentation have been documented in various studies. Thus, for example, Tomas et al. [28] reported that in two strains derived from *C*. *acetobutylicum* ATCC 824, genes of the fatty acid operon were down-regulated under butanol stress, whereas Wang et al. [30], when studying the same organism, observed up-regulation of fatty acid metabolism genes, under both butanol and butyrate stresses. Changes in membrane fluidity caused by the altered compositions of fatty acids cannot be the sole reason for the tolerance of microorganisms toward alcohols since most alcohol-tolerant organisms differ from intolerant ones in altered lipid contents that do not affect membrane fluidity to the same degree [31].

The phospholipid content, including their plasmalogen forms, of the obligate anaerobe genus *Clostridium* varies widely (see the Introduction). Based on our preceding analyses of lipids [32–34], we first performed a fast screening of polar lipids of *C*. *pasteurianum*. Lipidomic analysis by high resolution ESI on an Orbitrap mass spectrometer showed that, like other species of the genus *Clostridium*, *C*. *pasteurianum* also contains PE, PG, PS and CL as major lipid classes, both as diacyl and alkenyl (plasmenyl) acyl chains. In keeping with the literature data on other *Clostridium* species [14] the CL of *C*. *pasteurianum* also contained tetracyl, triacyl-plasmenyl and diacyl-diplasmenyl subclasses of CLs.

The highest concentration of membrane plasmalogens was reached approximately in the middle of exponential phase, when the cells produced butanol with the highest productivity. This correlates with the findings for *C*. *acetobutylicum* [21,35] that increased formation of plasmalogens, especially cardiolipin, reflects homeoviscous adaptation of cells under solvent-induced stress conditions. Cardiolipin changes in the cell membrane during solvent stress seem to be an important part of a complex cellular response [27].

In order to compare the stress response to butanol, either produced by the cells or added to the culture medium, we added 1-butanol to the bioreactor medium to a final concentration of 0.5% (v/v) (Fig. 1C and D).). This concentration was shown previously to be non-lethal for *C*. *pasteurianum* (data not shown). This pulse resulted in only a small reduction in growth rate, which was probably caused by induction of cellular adaptation, including the solvent stress response. As described above, one of the typical responses to butanol stress is the remodeling of membrane lipids. Surprisingly, butanol addition led to its next increase and also to an increase in the final butanol concentration at the end of cultivation. An unexpected activating effect of butanol (added to final concentrations of 2.5 or 7.5 g/l) on genes of the *sol* operon was described previously [28] for *C*. *acetobutylicum*.

The effect of added butanol is mainly reflected in the content of total plasmalogens. This is in contrast to the work carried out with the thermophilic ethanol producer *C*. *thermocellum*, in which a significant decrease in total DMA content was described in a solvent-tolerant strain, and in its cultivation with ethanol addition compared to the wild-type producer [29]. As described by Baer et al. [26], the addition of 1.0 or 1.5% (vol/vol) butanol to cells grown at 22 and 37°C caused an immediate (within 30 min) increase in the saturated/unsaturated FA ratio. Venkataramanan et al. [15] described similar increase in the saturated/usaturated FA ration after addition of butanol in various concentrations and during standard butanol fermentation in *C*. *pasteurianum* ATCC 6013. We also observed an increasing content of saturated FAs immediately (7^th^ hour of cultivation) after butanol addition (Table 2). Fig. 1B and 1D show that, at zero and six hours, the amount of total plasmalogens did not differ in cultivation with and without the addition of butanol. However, a rapid increase in their content occurred during sampling 60 min. after butanol addition (7^th^ hour of cultivation). However, additional samplings of both cultivations at the 12^th^ hour showed that the amounts of total plasmalogens in both cultivations were nearly equal.

Another change in lipid content concerned the ratio of saturated to unsaturated FAs. Vollherbst et al. [9] observed an increase in the total saturated to unsaturated FAs ratio after butanol addition, but as the concentration of palmitoleic acid increased with time of cultivation, so also did the content of saturated FAs (concentration of added butanol was 0.5 and 1%). Baer et al. [26] did not observe any marked effect of 1% and 1.5% added butanol on the ratio of saturated to unsaturated FAs in the collection strain, *C*. *acetobutylicum* ATCC 824, whereas such an effect was observed in the SA-2 mutant of this strain.

We observed a difference between the effect of butanol produced by cells and butanol added to the medium. In a 12-h culture, in which butanol was produced by the cells, the ratio of saturated to unsaturated FAs was 50:17, whereas in a culture to which butanol was added, this was 58:16. An even more striking change was observed in the 7^th^ hour, when the ratio in the latter culture was 69:8. It can therefore be inferred that when butanol is produced by the cells, changes in lipid content are not as striking as those caused by its addition, i.e. a steep increase in butanol concentration produces a strong shock and cells react to it rapidly (within 30 minutes).

## Supporting Information

S1 Fig The Orbitrap low resolution MS^2^ spectrum of c-p-19:0/17:1 PE (plasmalogen phosphatidyl ethanolamine). (TIF)

S2 Fig The Orbitrap low resolution MS^2^ spectrum of c-p-19:1/16:0 PG (plasmalogen phosphatidyl glycerol). (TIF)

S3 Fig The Orbitrap low resolution MS^2^ spectrum of c-p-19:1/16:0 PS (plasmalogen phosphatidyl serine). (TIF)

S4 Fig The Orbitrap low resolution MS^2^ spectrum of c-p-19:0/16:0/16:0/c-p-19:0 CL (diplasmalogen phosphatidyl *bis*-phosphatidyl glycerol). Structures of ions “a” and “b”, see S6 Fig.
(TIF)

S5 Fig The Orbitrap low resolution MS^3^ spectrum of c-p-19:0/16:0 CL, i.e. ion at *m/z* 813.7 from MS^2^ spectrum, see S4 Fig. (diplasmalogen phosphatidyl *bis*-phosphatidyl glycerol). Structures of ions “a” and “b”, see S6 Fig.
(TIF)

S6 Fig Presumed structures of ions “a” and “b”. (TIF)
