# Supplementary figures and images for: Changes in Membrane Plasmalogens of Clostridium pasteurianum during Butanol Fermentation as Determined by Lipidomic Analysis
Source: PLoS One. 2015 Mar 25;10(3):e0122058. doi: 10.1371/journal.pone.0122058 (PMC4373944; doi:10.1371/journal.pone.0122058)

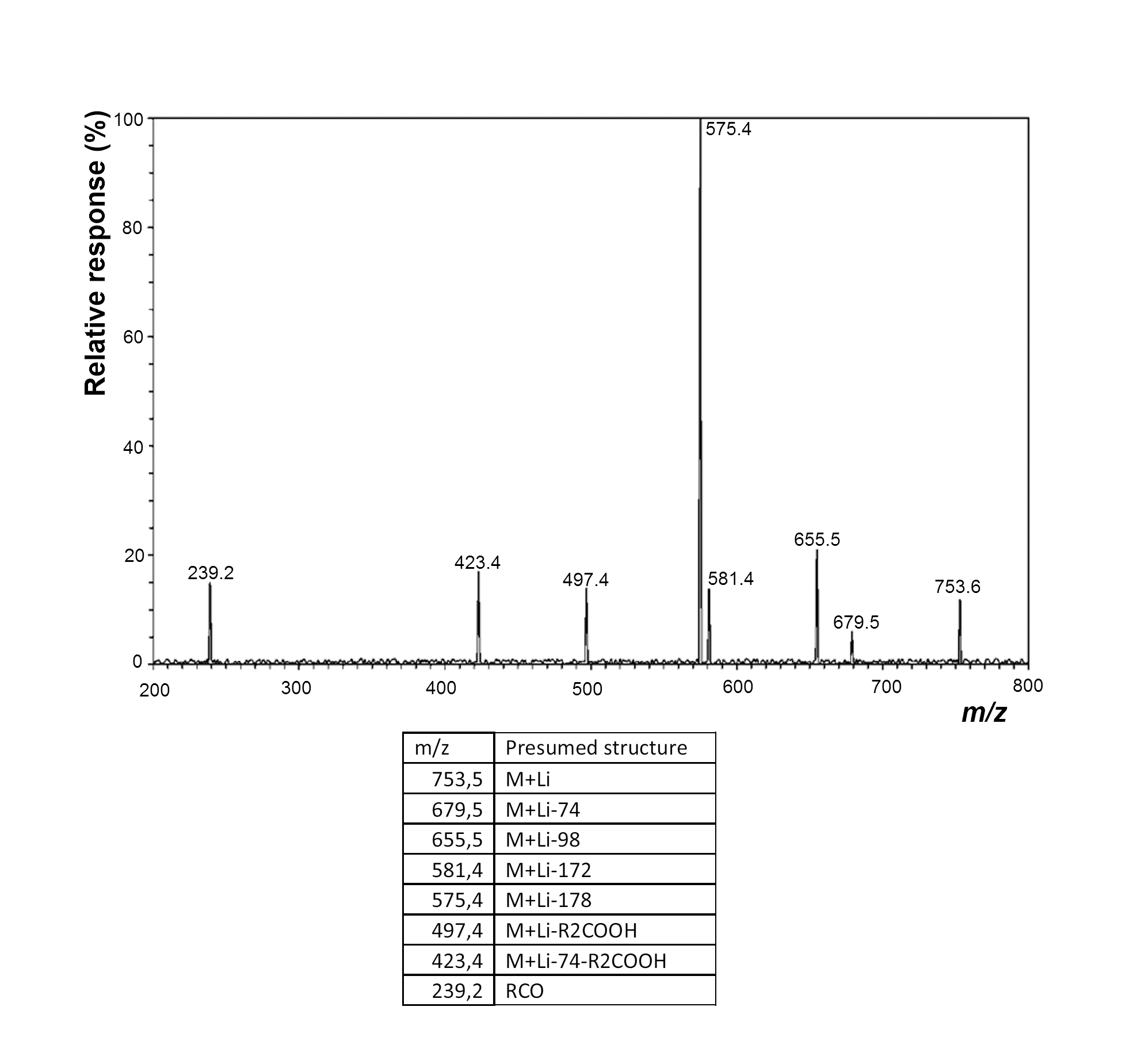

Supplement: S1 Fig — (TIF) [file pone.0122058.s001.tif]

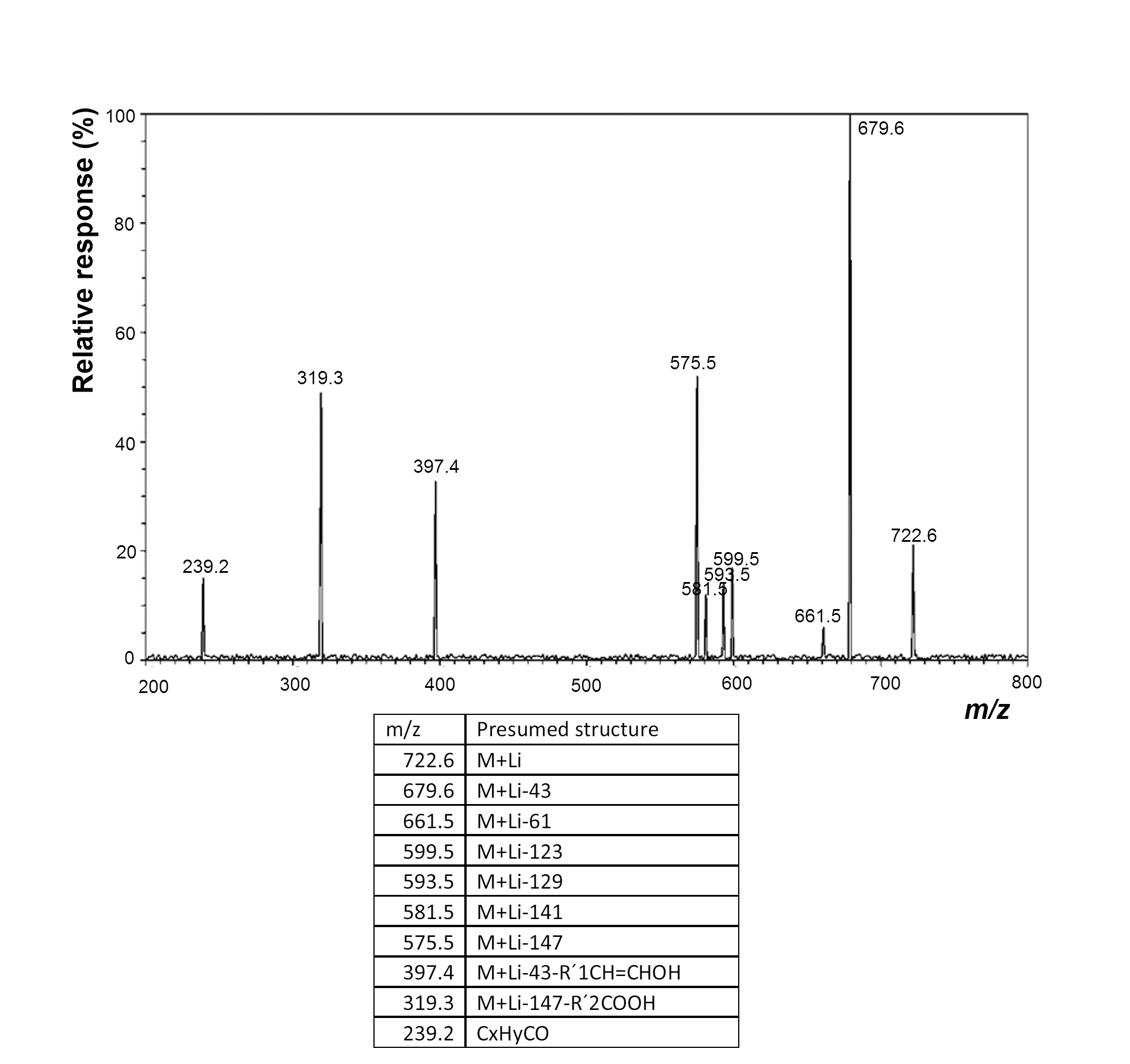

Supplement: S2 Fig — (TIF) [file pone.0122058.s002.tif]

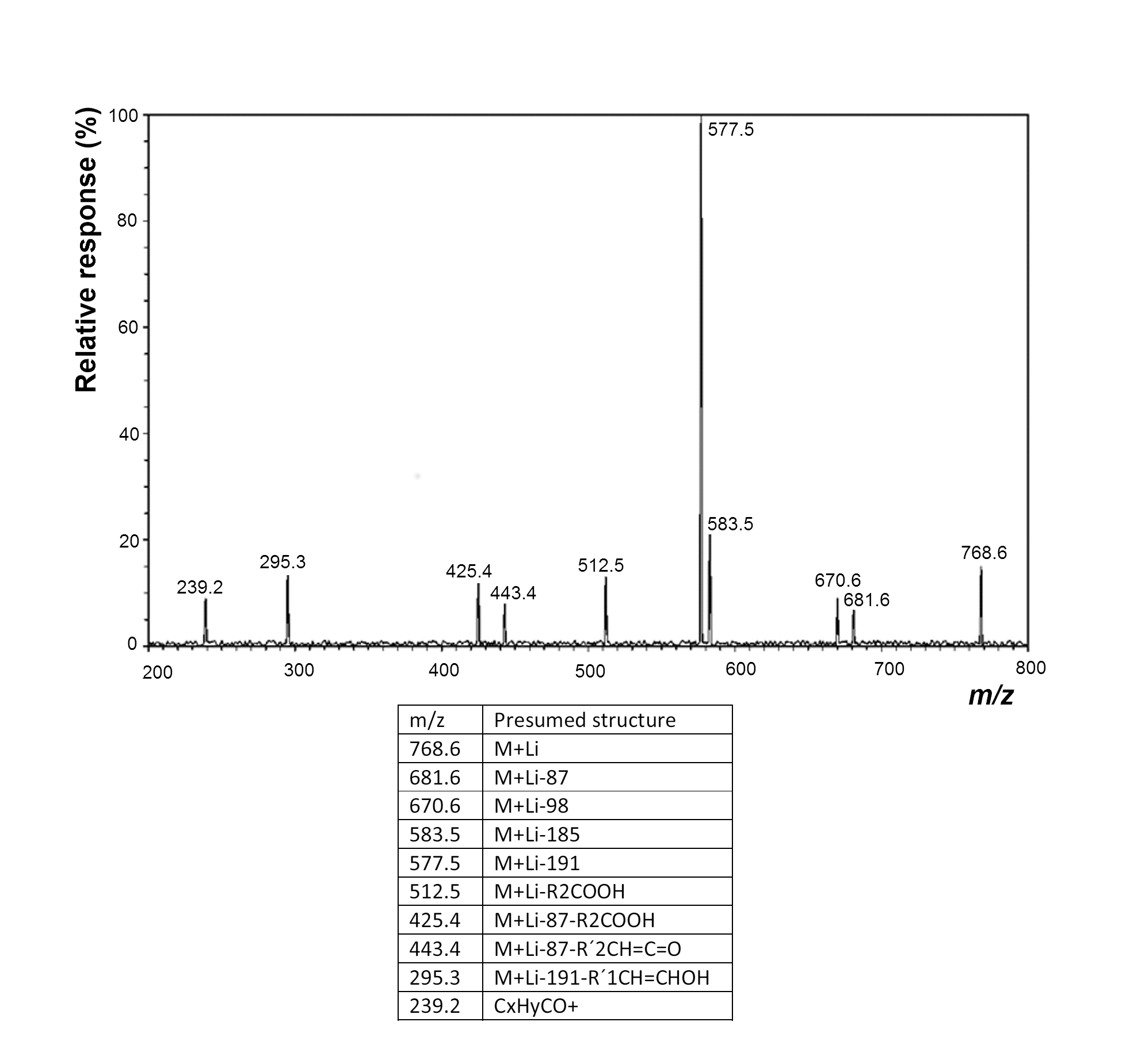

Supplement: S3 Fig — (TIF) [file pone.0122058.s003.tif]

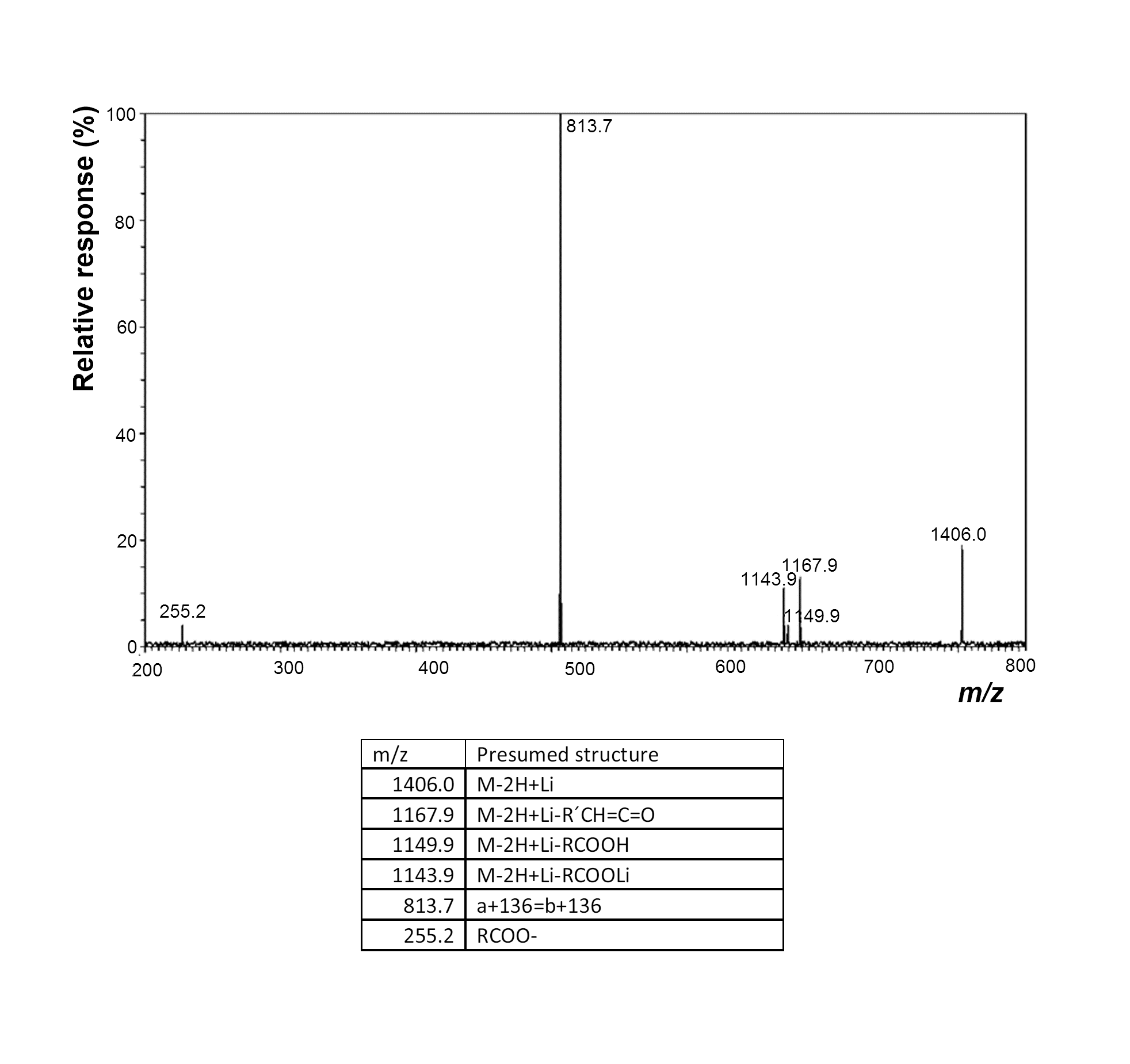

Supplement: S4 Fig — Structures of ions “a” and “b”, see S6 Fig. (TIF) [file pone.0122058.s004.tif]

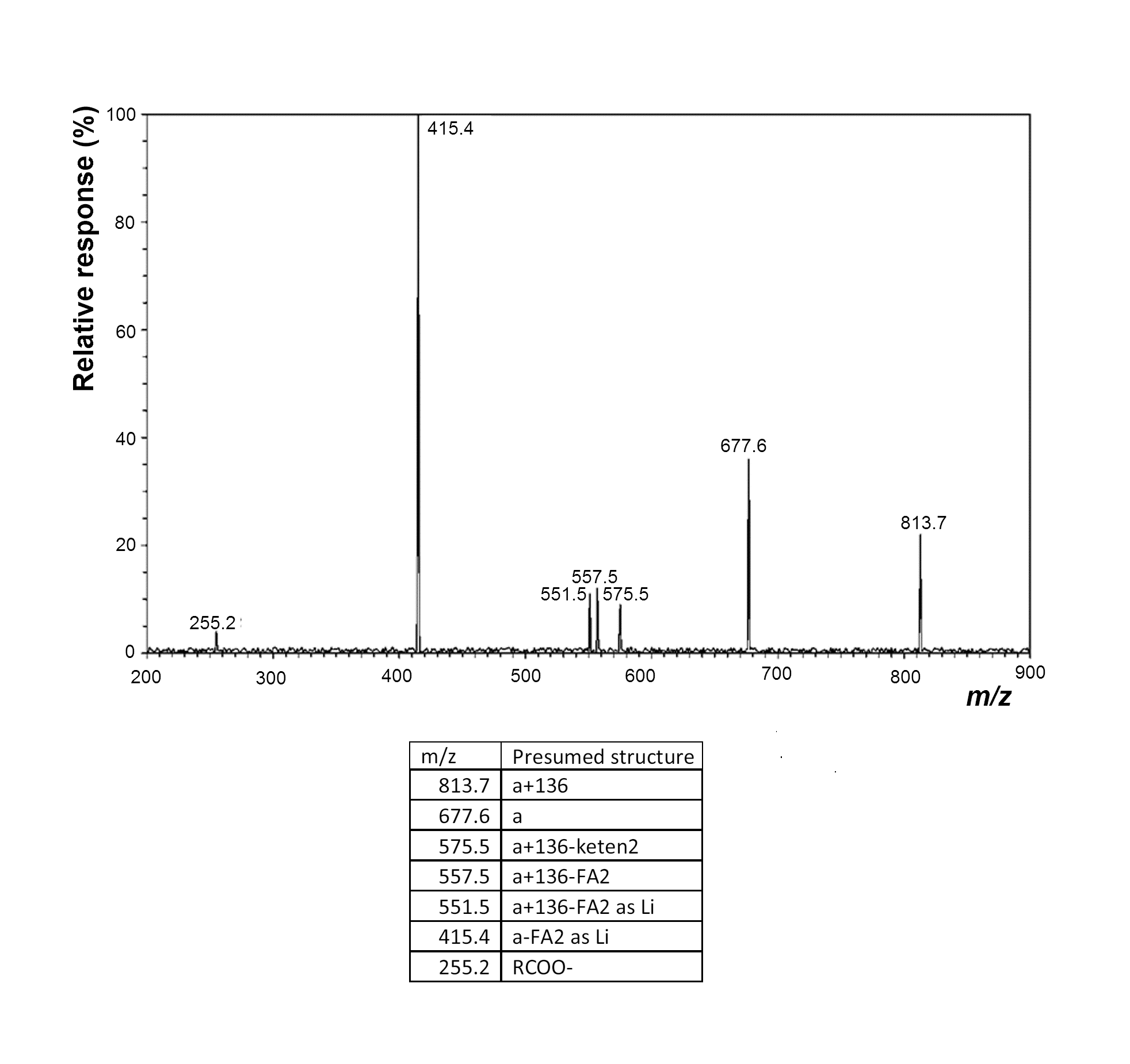

Supplement: S5 Fig — Structures of ions “a” and “b”, see S6 Fig. (TIF) [file pone.0122058.s005.tif]

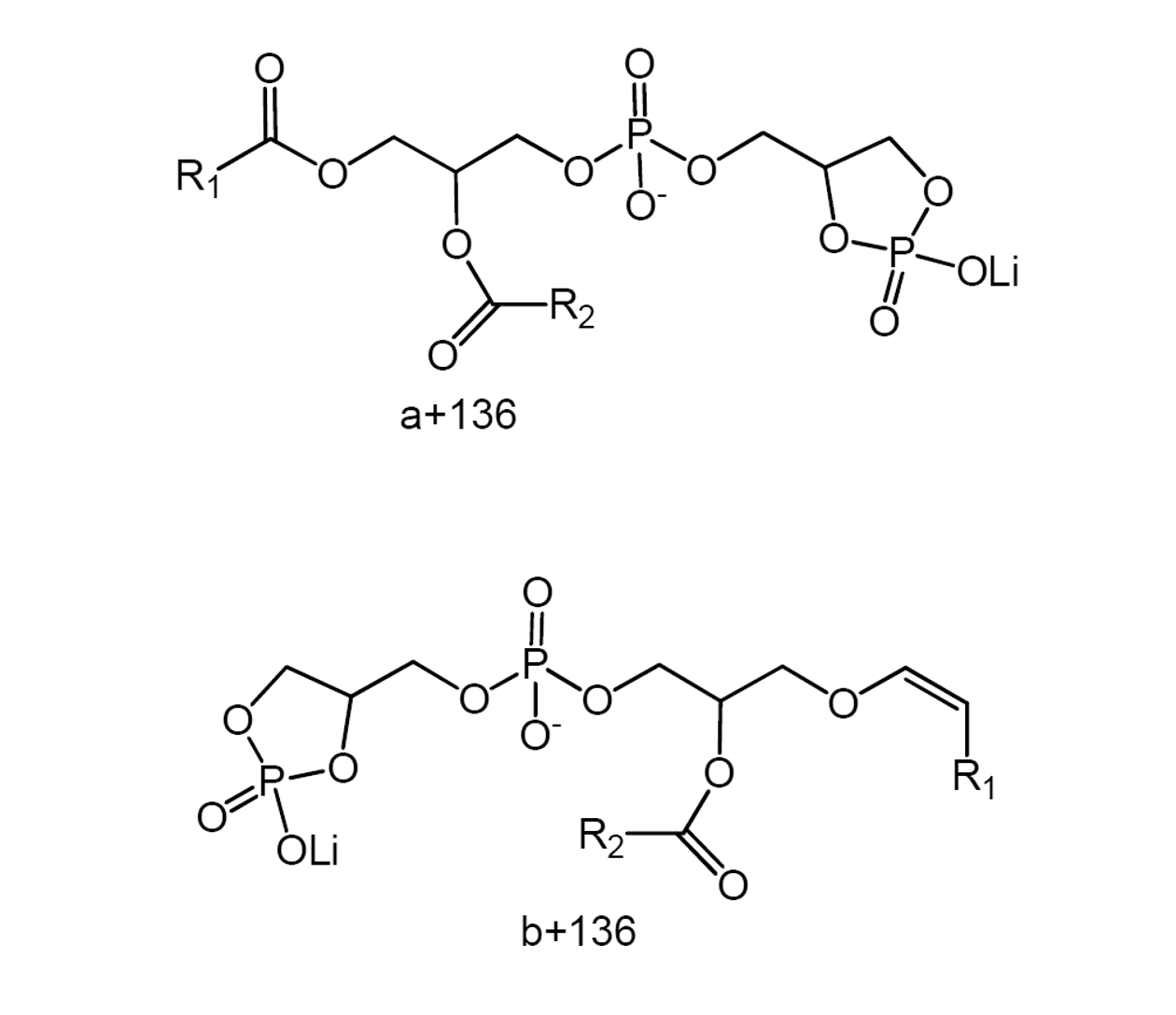

Supplement: S6 Fig — (TIF) [file pone.0122058.s006.tif]
